# Complement C3d Conjugation to Anthrax Protective Antigen Promotes a Rapid, Sustained, and Protective Antibody Response

**DOI:** 10.1371/journal.pone.0001044

**Published:** 2007-10-17

**Authors:** Ravi V. Kolla, Suresh Chintalapati, Mojgan Sabet, Eugenio Santelli, Robert C. Liddington, Michael David, Joshua Fierer, Donald Guiney, Robert C. Rickert

**Affiliations:** 1 Infectious and Inflammatory Disease Center, Burnham Institute for Medical Research, La Jolla, California, United States of America; 2 Division of Infectious Diseases, Department of Medicine, University of California at San Diego, La Jolla, California, United States of America; 3 Division of Biological Sciences, University of California at San Diego, La Jolla, California, United States of America; 4 Department of Medicine, San Diego Veterans Affairs Medical Center, San Diego, California, United States of America; Centre for DNA Fingerprinting and Diagnostics, India

## Abstract

*B. anthracis* is the causative agent of anthrax. Pathogenesis is primarily mediated through the exotoxins lethal factor and edema factor, which bind protective antigen (PA) to gain entry into the host cell. The current anthrax vaccine (AVA, Biothrax™) consists of aluminum-adsorbed cell-free filtrates of unencapsulated *B. anthracis*, wherein PA is thought to be the principle target of neutralization. In this study, we evaluated the efficacy of the natural adjuvant, C3d, versus alum in eliciting an anti-PA humoral response and found that C3d conjugation to PA and emulsion in incomplete Freund's adjuvant (IFA) imparted superior protection from anthrax challenge relative to PA in IFA or PA adsorbed to alum. Relative to alum-PA, immunization of mice with C3d-PA/IFA augmented both the onset and sustained production of PA-specific antibodies, including neutralizing antibodies to the receptor-binding portion (domain 4) of PA. C3d-PA/IFA was efficacious when administered either i.p. or s.c., and in adolescent mice lacking a fully mature B cell compartment. Induction of PA-specific antibodies by C3d-PA/IFA correlated with increased efficiency of germinal center formation and plasma cell generation. Importantly, C3d-PA immunization effectively protected mice from intranasal challenge with *B. anthracis* spores, and was approximately 10-fold more effective than alum-PA immunization or PA/IFA based on dose challenge. These data suggest that incorporation of C3d as an adjuvant may overcome shortcomings of the currently licensed aluminum-based vaccine, and may confer protection in the early days following acute anthrax exposure.

## Introduction

Anthrax, an acute infectious disease caused by the spore-forming bacteria *B. anthracis*, has come to recent prominence as an agent of bioterrorism. Acute exposure to anthrax can be treated effectively with antibiotics, passive antibody transfer and newly developed biological or chemical inhibitors of anthrax toxin. However, protection from future exposure or germination of residual spores can only be attained by vaccination and the elicitation of a strong humoral response. The currently licensed anthrax vaccine (Biothrax™) is a cell-free filtrate derived from an avirulent unencapsulated strain of *B. anthracis* and adsorbed to an aluminum hydroxide salt (alum). While limited case studies indicate that Biothrax™ is protective, concerns remain regarding efficacy and safety [1]. Approximately 30% of patients who are vaccinated with Biothrax™ suffer from mild skin irritation and cutaneous-anthrax-like symptoms, which are thought to be due to toxins present in the filtrate [2], [3], [4]. The vaccination regimen requires six injections over the course of 18 months and frequent boosters to achieve and maintain full effectiveness [4], [5]. Therefore, there is an acknowledged need for an improved vaccine that exhibits prompt and sustained effectiveness and is free of reactogenic properties [6]. Specifically, next generation anthrax vaccines aim to: 1) induce rapid neutralizing antibody titers, 2) maintain high titers of neutralizing antibody, 3) induce robust memory responses, and 4) be free of reactogenic properties that may cause illness or discomfort. In addition, vaccines for use in the general public need to impart protection in infants and the elderly.

The major virulence factors of *B. anthracis* provide the most promising targets for vaccine development and are encoded by 2 plasmids, pXO1 and pXO2. Plasmid pXO1 encodes the toxin components lethal factor (LF), edema factor (EF) and protective antigen (PA), which work collaboratively to promote anthrax toxicity [7], [8], [9]. Plasmid pXO2 encodes the capsule carbohydrate poly-γ-D-glutamic acid that prevents phagocytosis, but is not toxigenic. Toxin pathogenesis is initiated by PA binding to TEM8 or CMG2 on the macrophage cell surface to allow heptamerization of PA and subsequent association with LF or EF [7], [10]. The PA heptamer and a single LF/EF molecule form a large complex which induces receptor-mediated internalization into endocytic vesicles that mature into acidic lysosomes. Low pH within the vesicle changes the conformation of PA to create a pore that allows exit of LF or EF into the cytoplasm before degradation can occur [8]. Edema toxin (EdTx), the combination of PA and EF, primarily affects neutrophil function by disregulating water homeostasis leading to edema [8]. Lethal toxin (LeTx), the combination of PA and LF, causes cleavage of MAPK family members leading to apoptosis of the infected cell [8]. In mouse, macrophage sensitivity to LeTx-induced apoptosis does not always correlate with strain susceptibility to anthrax infection, indicating that additional understanding of the pathophysiology is needed.

Protective antigen is so named for its ability to elicit a protective immune response to anthrax infection, consistent with evidence that protection provided by Biothrax™ vaccination is attributed to PA-specific antibodies [1], [4]. Protective antigen has no intrinsic enzymatic activity or pathogenic function, but is essential for the cellular entry of LF and EF. Disruption of domain 4 (amino acids 587–735) of PA completely abrogates binding to cell surface receptors and consequently negates toxin pathogenicity [11], [12]. With the intent of curtailing side effects of Biothrax™ and eliciting a more robust PA-specific IgG response, one promising new approach for an improved vaccine is to utililize recombinant PA (rPA) instead of cell-free filtrates [1].

The only approved adjuvants in the U.S. contain aluminum hydroxide or phosphate salts (alum) and therefore much of the work in testing new anthrax vaccine targets have used alum [13], [14], [15]. Correspondingly, relatively little attention has been paid to the development of alternative adjuvants to augment antigen-specific B cell responses. In seminal work, Dempsey *et al* reported the use of C3d, a breakdown product of complement serum protein C3, as a natural molecular adjuvant that significantly augmented antigen-specific antibody titers [16]. C3d has since been used in mice for a variety of vaccine applications including HIV, measles, *S. pneumoniae* and influenza [17], [18], [19], [20], [21], [22]. C3d function is primarily mediated through its binding to complement receptors CD21/CD35 expressed on both B cells and follicular dendritic cells (FDCs). Though the mechanism of C3d function has not been fully elucidated, work from our group and others suggests that binding of complement receptors leads to retention of antigen on the FDC or B cell surface and prolongs signaling by the B cell receptor complex, leading to augmented B cell activation [23], [24], [25], [26], [27], [28], [29].

In this study, we evaluated the use of C3d as an adjuvant for PA. We demonstrate that conjugation of PA to trimeric C3d (C3d_3_) in the presence of IFA induces a more robust and protective IgG response to intact PA or domain 4 as compared to PA in IFA or PA adsorbed to alum (PA-Alum). The response elicited by C3d conjugation occurs more rapidly and is at least as durable as PA-alum after a single immunization. Furthermore, C3d_3_-PA is effective when administered by various routes of immunization and is functional in adolescent mice. Lastly, we find that increased efficacy of C3d_3_-PA/IFA may derive in part from its ability to augment the germinal center (GC) response and enhance B cell differentiation into plasma cells and memory B cells.

## Materials and Methods

### Mice

6–8 week old A/J, C3H/HeJ and BALB/c mice were purchased from Jackson laboratories (Bar Harbor, ME). All animals were maintained in a pathogen-free environment and handled in accordance with the guidelines set forth by NIH and animal subjects programs at the Burnham Institute for Medical Research or UCSD.

### Purification of C3d_3_-bio and conjugation to PA

Cloning of murine *C3d* and expression of the biotinylated form (C3d_3_-bio) was described previously [29]. Briefly, pXA3-C3d_3_-bio was transformed into BL21A1 cells and cultures grown in Luria broth (supplemented with 50 µg/mL carbenicillin and 0.1% glucose) to an OD of 0.4–0.6 at 30°C. C3d_3_-bio expression was induced by the addition of arabinose (0.1%) and IPTG (0.5 mM). Overexpressed protein was purified using Ni-NTA beads (Qiagen, Valencia, CA), dialyzed against PBS, concentrated (Amicon ultra; Millipore, Billerica, MA) and treated for endotoxin removal (EndoClean™; BioVintage, San Diego, CA). Protective antigen (BEI Resources, NIAID, Bethesda, MD) was *in vitro* biotinylated (EZ link Sulfo-NHS-biotin; Pierce, Rockford, IL), subjected to endotoxin removal and conjugated with C3d_3_-bio at a molar ratio of 1∶2.5 using an avidin bridge (Sigma, St. Louis, MO; hen egg avidin expressed in plant and admixed with an equimolar amount of PA).

### Expression of PA and domain 4

DNA segments encoding full length PA (for coating of ELISA plates) or domain 4 (amino acids 587–735) were cloned into pET15b and expressed in BL21 DE3 cells. Cells were grown to an OD of ∼0.5 at 37°C and the culture induced with 0.2 mM IPTG for 4 hrs after shifting and equilibrating the culture for 15 min at 30°C. Overexpressed proteins were affinity purified using Ni-NTA agarose beads (Qiagen) binding the C-terminal His tag in both proteins.

### Immunizations

Mice received PA-Av, PA-Av-C3d [both emulsified 1∶1 in IFA (Sigma)] or PA-Av adsorbed to alum [ratio of 1∶1], (Imject; Pierce). Injection volume was 200 µl when injected i.p., 100 µl when injected subcutaneously (s.c) and 50 µl for intramuscular (i.m.) injections. For i.m. injections, PBS was used instead of IFA. All mice were immunized with either 10 or 20 µg of PA, as noted in the Results. All secondary immunizations were given the respective amount of PA in PBS i.p. Immunizations of adolescent mice were performed on groups of 2–3, 21–25 day old A/J mice. These mice received 10 µg PA and were injected i.p. The kinetics of GC formation and accumulation of plasma cells and activated T cells were assessed by flow cytometry on immunized groups of 3 mice each sacrificed at 0, 2, 4, 8, or 12 days post-immunization.

### ELISA

Peripheral blood was isolated from mice by retro-orbital bleed before immunization and at 7–10 day intervals after immunization. Serum antibody titers against PA or domain 4 were detected using 96 well plates (BD Biosciences, San Diego, CA) coated with either 10 µg/ml PA or domain 4 and developed with alkaline phosphatase-conjugated rat anti-mouse IgG, IgM, IgG1, IgG2a, IgG2b or IgG3 secondary antibodies (Southern Biotechnology, Birmingham, AL). PNPP substrate (Southern Biotechnology) was used for colorimetric quantification at 405 nm on a microplate reader (ELx808, Bio-Tek, Winooski, VT). For comparisons among animals, single point dilutions were selected following the determination of the linear range of serum antibody binding.

### Flow cytometry

Single cell spleen cell suspensions were prepared and red blood cells removed by ACK lysis. 1.5×10^6 ^cells were analyzed per staining reaction and analyzed using a FACSCanto (BD Biosciences). Stains for germinal center B cells included B220-APC-Cy7, IgD-bio, Sav-Pcp-Cy5.5, CD3-PE-Cy7, GL7-fitc, Fas-APC, CD11b-PE (BD Pharmingen, eBiosciences, San Diego, CA). Stains for plasma cells substituted Syn1-PE, IgD-fitc, IgM-APC, and CD3-fitc. T cell activation stains included ICOS-PE, CD62L-APC, CD4-APC-Cy7 and B220-APC.

### Histology

Spleens were harvested and placed in O.C.T. (Tissue-Tek®, Sakura Finetek, Torrance, CA) followed by snap-freezing in liquid nitrogen. Seven µM sections were prepared and stained and stained with combinations of B220-APC, CD4-FITC, IgM-APC, IgD-FITC, CD35-bio, GL7-FITC (BD-pharmingen; eBioscience). Images were acquired using a Zeiss Axiocam M1 microscope/10X objective and Slidebook software (Intelligent Imaging Innovations, Denver, CO).

### Mouse challenge with B anthracis

Spores were prepared from the Sterne strain of *B. anthracis* (pXO1^+^pXO2^−^) as previously described [30]. Prior to infection, the spores were heated to 65°C for 30 min to activate spores for germination. Groups of female A/J mice were immunized i.p. as described above and challenged 10 days later by intranasal administration of 20 µl of the *B. anthracis* spore preparation containing the indicated inoculum. Survival was monitored for 10 days after infection.

## Results

### C3d_3_ conjugation to PA is superior to alum adsorption in eliciting antibody responses to intact PA and domain 4

**Figure 1:**
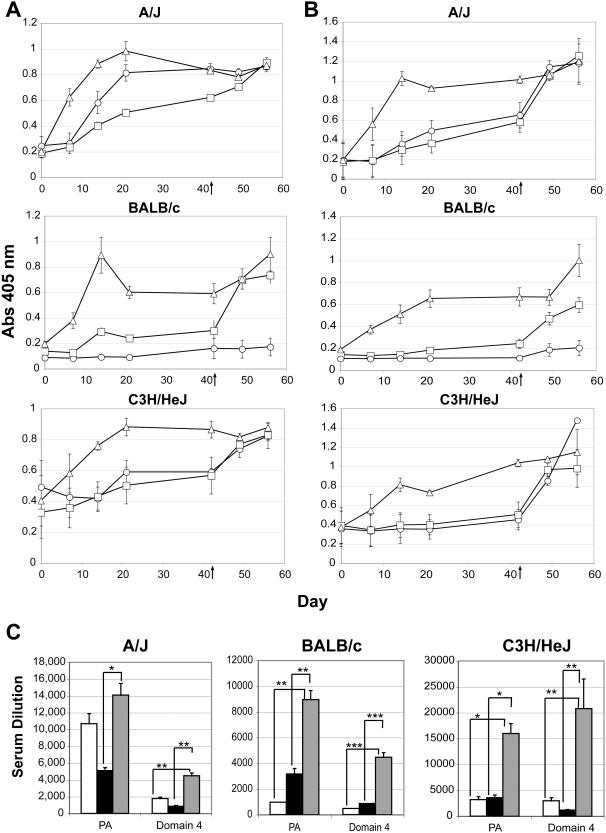
Immunization with PA-Av-C3d_3_/IFA augments production of PA- and Domain 4-specific antibodies relative to alum. Groups of A/J (top), BALB/c (middle), and C3H/HeJ (bottom) mice were immunized with PA-Av/IFA (○), PA-Av-Alum (□), or PA-Av-C3d_3_/IFA (▵). (A) Serum PA- and (B) Domain 4-specific IgG was measured by ELISA (Abs 405 nm). All mice were boosted at day 42 (↑) with PA in saline. Error bars indicate SEM (n = 5). (C) Endpoint ELISAs were performed on day 14 serum samples from PA-Av/IFA, PA-Av-Alum, and PA-Av-C3d_3_/IFA immunized mice. Endpoint titers shown are 2-fold above background. Error bars represent SEM (n = 5, *p≤0.05, ** p≤0.01, *** p≤0.005).

To determine if C3d_3_ is an effective adjuvant for PA vaccination, we compared B cell immune responses in mice from several strains immunized with: PA-alum, PA-C3d_3_/IFA or PA in IFA. For preparation of the immunogen, full length (83 kD) PA was *in vitro* biotinylated and either conjugated to biotinylated-C3d_3_ via an avidin (Av) bridge (PA-Av-C3d_3_) and emulsified in IFA, complexed with Av and adsorbed to alum (PA-Av-Alum) or complexed in the presence of Av and emulsified in IFA. Groups of A/J, BALB/c, and C3H/HeJ mice were immunized intraperitoneally (i.p.) with equivalent amounts (20 µg) of PA and PA-specific IgG measured by ELISA. In all strains of mice, PA-Av-C3d_3_/IFA elicited a markedly greater IgG response than PA-Av-Alum or PA/IFA during the primary response (Figure 1A). Elevated titers of PA-specific IgG were observed by 7 days after initial immunization with PA-Av-C3d_3_/IFA - approximately 2.5- to 3-fold enhanced over PA-Av-Alum immunization. Peak IgG titers were observed between days 14 and 21 in PA-Av-C3d_3_/IFA immunized mice. At day 42, all groups received a boost of PA in saline. Mice receiving PA-Av-Alum showed a clear secondary response, indicating that although alum did not provide a robust primary IgG response, it was capable of priming a secondary response. In contrast, secondary immunization of PA-Av-C3d_3_/IFA-primed mice with soluble PA maintained the elevated specific IgG titers observed during the primary immunization. High levels of PA-specific titers remained in the serum of C3d-immunized mice for over 60 days post-secondary immunization (data not shown). Results from the immunization of C3H/HeJ mice also demonstrated that preparation of the C3d adjuvant was free of LPS endotoxin contamination as these mice express a nonfunctional TLR4 protein [31]. Curiously, PA-Av/IFA elicited strong IgG responses in A/J mice in both the primary and secondary responses. However, PA/IFA alone (non biotinylated, no Av) elicited similar IgG responses (data not shown), and BALB/c and C3H/HeJ mice did not respond well to PA-Av/IFA. Therefore, we conclude that A/J is a high-responder strain to PA, and that the C3d-adjuvant effect is not due to multimerization of PA or the use of IFA (data not shown). Most Importantly, C3d elicited a consistent 2.5-3 fold greater primary antibody response as compared to alum, regardless of the mouse strain used.

Antibodies specific for domain 4 of PA are capable of neutralizing toxin by blocking binding of the toxin to host cell surface receptors [10], [32], [33]. Indeed, passive protection from anthrax exposure is conferred by injection of monoclonal antibody 14B7, which binds to this region with high affinity [32]. We therefore tested whether immunization with PA-Av-C3d_3_/IFA efficiently produced domain 4-specific antibodies (Figure 1B). In all strains of mice tested (A/J, BALB/c, and C3H/HeJ), primary immunization with PA-Av-C3d_3_/IFA elicited an approximately 3-fold enhancement in domain 4-specific IgG titers relative to PA-Av-Alum or PA-Av/IFA (Figure 1B). Secondary immunization of A/J and C3H/HeJ mice with soluble PA alone did not further elevate the existing high titers of domain 4-specific antibody present in PA-Av-C3d_3_/IFA-immunized mice. However, BALB/c mice did show a modest increase in domain 4-specific IgG after the boost (Figure 1B).

To quantitate antigen-specific titers in PA-Av-C3d_3_/IFA immunized mice, endpoint ELISAs were also performed on serum isolated during the peak primary response (day 14) from all immunized mice. In all strains of mice, we confirmed that PA-Av-C3d elicits a 3-5 fold increase in PA- or domain 4-specific antibodies as compared to PA-Av-Alum or PA/IFA (Figure 1C). Altogether, these data indicate that C3d_3_/IFA is a more effective adjuvant combination than alum in eliciting a rapid and sustained antibody response to PA and, in particular, neutralizing antibodies to domain-4 after a single immunization. The adjuvant effect is chiefly attributed to C3d rather than IFA, since emulsion of PA-Av in IFA does not elicit a significantly enhanced antibody response relative to PA-Av in PBS (data not shown).

### PA-Av-C3d_3_ augments production of isotype switched antibody

**Figure 2:**
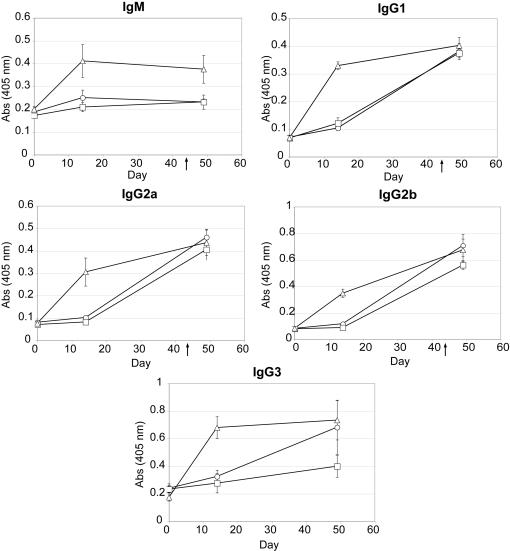
Immunization with PA-Av-C3d_3_/IFA augments production of isotype switched antibody. Domain 4-specific IgM, IgG1, IgG2a, IgG2b and IgG3 ELISAs (Abs 405 nm) were performed from A/J mice immunized with PA-Av/IFA (○), PA-Av-Alum (□), or PA-Av-C3d_3_/IFA (▵). All error bars indicate SEM (n = 5).

To determine adjuvant-dependent differences in generating neutralizing class-switched domain 4-specific antibodies, antigen-specific ELISAs were performed to measure IgM and IgG titers in A/J mice immunized with PA-Av/IFA, PA-Av-Alum or PA-Av-C3d_3_/IFA (Figure 2). We observed that C3d_3_-conjugation dramatically enhanced domain 4-specific IgM and all IgG subtypes during the primary response. At day 14, PA-Av-C3d_3_/IFA induced, at minimum, a 2-fold increase in serum IgM, IgG1, IgG2a, IgG2b and IgG3 titers as compared to PA-Av-Alum or PA-Av/IFA. However, following the boost at day 49, C3d_3 _adjuvant only increased serum titers of IgM and IgG3 relative to alum (Figure 2). A similar isotype distribution was observed for antibodies binding intact PA (data not shown).

### PA-Av-C3d is effective when administered via intraperitoneal or subcutaneous routes

**Figure 3:**
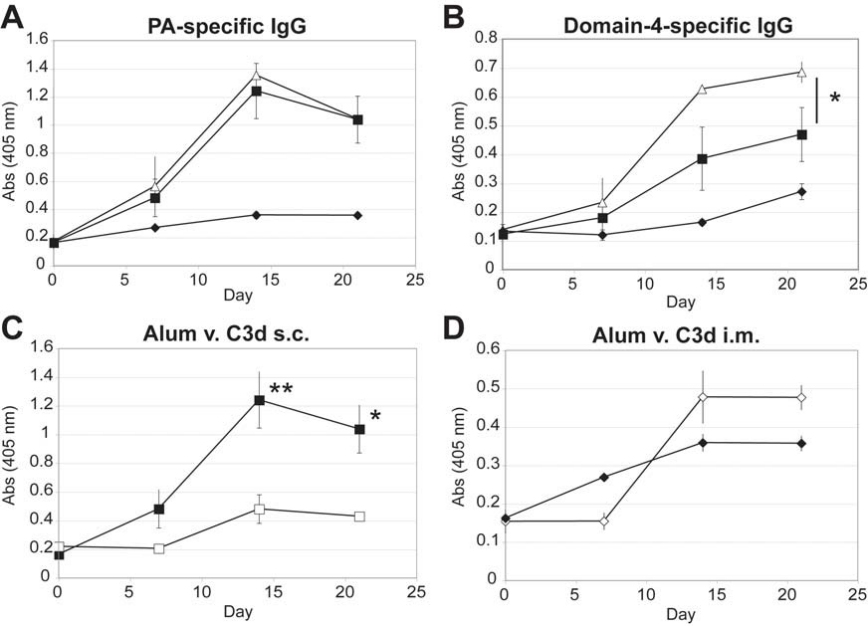
PA-Av-C3d_3_ is an effective immunogen delivered i.p., s.c or i.m. PA-Av-C3d_3_ was injected i.p. (▵), s.c. (▪), or i.m. (♦) and (A) PA- or (B) Domain 4-specific IgG was measured by ELISA. Relative efficacy of PA-Av-C3d_3_ (▪,♦) and PA-Av-alum (□,⋄) was compared following (C) s.c. or (D) i.m. administration of immunogen (see methods). Error bars indicate SEM (*p≤0.05, ** p≤0.01, *** p≤0.005, n = 3).

The adjuvant function of C3d is primarily mediated through its high affinity interaction with complement receptor CR2 (CD21/35) expressed on B cells and FDCs [34], [35]. However, B cells and FDCs are not likely to be present at sites of subcutaneous (s.c.) or intramuscular (i.m.) delivery, which are the sites of administration for the Biothrax™ and rPA102 vaccines [4], [36]. Therefore, we determined how delivery of C3d_3_ by these routes would compare with the potency observed after i.p. injection. Interestingly, we found that PA-Av-C3d_3_/IFA produced equivalent titers of anti-PA IgG antibodies when administered via i.p. or s.c. routes, but showed reduced effectiveness i.m. (Figure 3A). Similar findings were obtained for domain 4-specific IgG (Figure 3B). In comparative analyses, anti-PA titers from mice immunized s.c. with PA-Av-C3d_3_/IFA were elevated 3-fold relative to PA-Av-Alum (Figure 3C). Intramuscular immunization with PA-Av-C3d_3_ or PA-Av-Alum produced similar responses (Figure 3D). These data indicate that C3d retains its potency as an adjuvant when administered s.c, and is comparable to alum when injected i.m.

### PA-Av-C3d elicits strong antibody responses in adolescent mice

**Figure 4:**
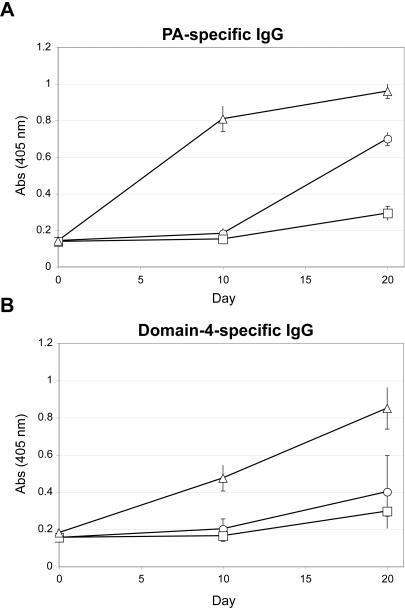
C3d is a functional adjuvant in adolescent mice. 3-week old A/J mice were immunized with PA-Av/IFA (○), PA-Av-Alum (□), or PA-Av-C3d_3_/IFA (▵). (A) PA-specific IgG or (B) domain-4 specific IgG was measured by ELISA up to 20 days after primary immunization. Error bars indicate average of 2 mice/group, and was repeated twice.

Another key parameter in vaccine development is safety and efficacy in infants and children. We therefore investigated whether C3d would be an effective adjuvant in adolescent mice, which lack a fully mature B cell compartment. In particular, marginal zone B cells (MZBs), which line the splenic marginal sinus and respond to blood born antigens, are underrepresented in the young. This is an important consideration since MZBs express high levels of CD21 and may be key mediators of C3d function [37], [38]. Characterization of B cell compartments in 3-week old A/J mice showed an over-abundance of immature-mature “transitional” B cells in the spleen; however follicular structure and B/T segregation was intact (data not shown). As expected, MZBs were present, but at a lower frequency than in adult mice (data not shown). Twenty-two day old A/J mice were immunized i.p. with equivalent amounts of PA (10 µg) in the form of PA-Av/IFA, PA-Av-Alum, or PA-Av-C3d_3_/IFA. Serum was collected every 10 days and PA- and domain 4-specific IgG titers measured by ELISA (Figure 4A,B). As seen in adult mice, PA-Av-C3d_3_/IFA elicited greater PA- and domain 4-specific IgG than PA-Av/IFA or PA-Av-Alum (Figure 4A,B). The kinetics of the response was also similar to adult mice insofar as significant levels of IgG were detected by day 10 and increased through day 20 (Figure 4A,B). Conversely, alum-immunized mice mounted minimal PA- and domain 4-specific antibody responses. PA-Av/IFA also induced PA-specific IgG; however minimal domain 4-specific IgG was detected. Thus C3d_3_ is an effective adjuvant in adolescent mice.

### C3d_3_ augments immune responses by abetting the formation of germinal centers and plasma cell differentiation

**Figure 5:**
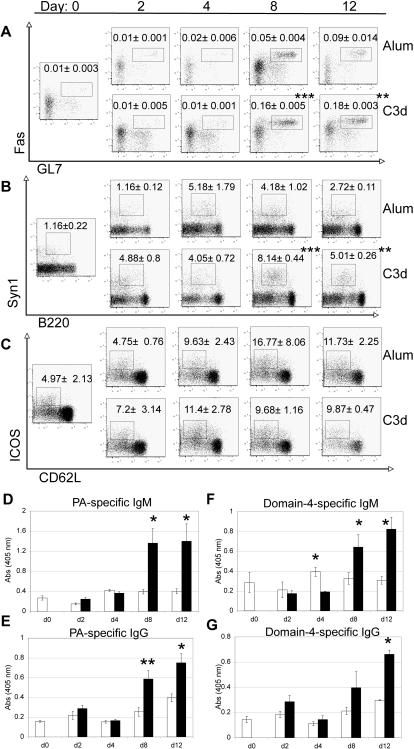
C3d conjugation augments the formation of germinal centers and plasma cells. A/J mice were analyzed at the indicated time points post-immunization with PA-Av-Alum or PA-Av-C3d_3_/IFA. Flow cytometry profiles indicate (A) percent splenic germinal center B cells (CD3^−^, CD11b^−^, IgD^−^, B220^+^, GL7^+^, Fas^+^) of total B cells; (B) percent plasma cells (CD11b^−^, CD3^−^, IgD^−^, B220^lo^, Syn1^hi^) of IgD^−^ B cells, and (C) percent activated T cells (B220^−^, CD11b^−^, CD4^+^, CD62L^lo^, ICOS^+^) of CD4^+^ cells. Representative FACS plots are shown with average and standard deviation (n = 3) of gated population indicated. ELISAs were performed to measure PA-specific (D) IgM and (E) IgG and domain 4-specific IgM (F) and IgG (G) from serum of mice immunized with PA-Av-Alum (white columns) or PA-Av-C3d_3_/IFA (black columns). Error bars represent SEM (n = 3) (*p≤0.05, **p≤0.01, ***p≤0.005).

Germinal centers (GCs) are specialized structures within follicles wherein B cells undergo somatic hypermutation and class switch recombination of rearranged immunoglobulin genes to generate high-affinity plasma cells or memory B cells [39]. To address how C3d_3_ enhances the antibody response, we investigated the effect of C3d conjugation versus alum adsorption on the formation of GCs and plasma cells. Groups of A/J mice were immunized with PA-Av-Alum or PA-Av-C3d_3_/IFA and analyzed by flow cytometry and immunohistology at 0, 2, 4, 8 and 12 days post-immunization to enumerate splenic GCs, plasma cells and activated T cells. Serum was collected from all mice and PA- and domain 4-specific IgG titers determined by ELISA (Figure 5).

Analysis of PA–Av-Alum- and PA-Av-C3d_3_/IFA-immunized mice showed that immunization with C3d_3_ enhanced the generation of GL7^+^, Fas^+^ GC B cells (Figure 5A). Peak GC B cell production occurred on day 8 (0.16%) in PA-Av-C3d_3_/IFA-immunized mice and was approximately 3-fold greater than in PA-Av-Alum-immunized mice (0.05%) (Figure 5A). At day 12, differentiation into GC B cells was increased in PA-Av-Alum-immunized mice, while PA-Av-C3d_3_/IFA-immunized counterparts maintained the high percentage of GC B cells seen at day 8. Immunofluorescent staining of spleen sections corroborated the flow cytometric data and showed that PA-Av-C3d_3_/IFA immunization elicited larger and more frequent GCs than PA-Av-Alum-immunized counterparts (data not shown). Although PA-Av/IFA immunizations were not included in this experiment, these findings in conjunction with the PA-specific IgG responses shown in Figure 1 support the conclusion that C3d_3_ conjugation hastens the onset and promotes the maintenance of the GC reaction.

In addition to GC formation, C3d_3_ conjugation also promoted plasma cell generation more efficiently than alum, as evidenced by the increased (4-fold) generation of B220^lo^, Syn1^hi^ B cells as soon as 2 days post-immunization (Figure 5B). Peak plasma cell formation in PA-Av-Alum immunized mice (5.2%) was observed at day 4 after immunization, whereas PA-Av-C3d_3_/IFA induced peak plasma cell levels at day 8 (8%) (Figure 5B). Furthermore, a high percentage of plasma cells was maintained through day 12 in PA-Av-C3d_3_/IFA immunized mice. Determination of serum PA- and domain 4-specific IgM and IgG titers corroborated the plasma cell data as PA-Av-C3d_3_/IFA immunization induced a 3-fold increase in PA-specific IgM and IgG at day 8 as compared to alum (Figure 5D,E). Domain 4-specific IgM and IgG showed a significant 2-fold increase at day 8 (Figure 5F,G). These data are consistent with the magnitude and kinetics of the anti-PA/domain 4 responses shown in Figure 1.

Analysis of *in vivo* T cell activation in PA-Av-C3d_3_/IFA and PA-Av-Alum immunized mice revealed similar percentages of activated T cells (CD62L^lo^, ICOS+) (Figure 5C), though the subset of PA-specific T cells was not assessed. We speculate that the augmented GC reaction and plasma cell formation is through direct B cell activation by co-ligation of C3d_3_ and the BCR, as well as CD21-facilitated uptake of antigen for presentation to T cells. No evidence of elevated TNFα, IL1-β or IL-6 was observed in the serum of PA-Av/IFA, PA-Av-Alum, or PA-Av-C3d_3_/IFA immunized mice at 1.5, 6, or 24 hours after immunization nor at any of the time points shown in Figure 5, indicating that C3d_3_ is not generally pro-inflammatory (data not shown).

### PA-Av-C3d_3_ provides enhanced protection against aerosol anthrax spore challenge

**Figure 6:**
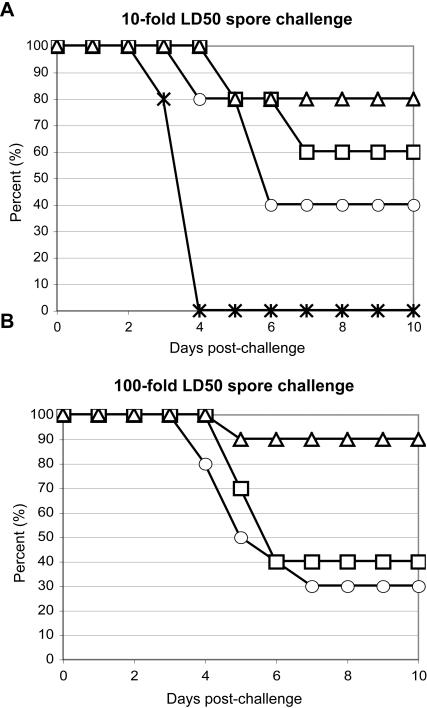
PA-Av-C3d_3_ provides enhanced protection against aerosol anthrax spore challenge. A/J mice were immunized with either: adjuvant control (*), PA-Av/IFA (○), PA-Av-Alum (□), or PA-Av-C3d_3_/IFA (▵). Ten days after immunization, mice were challenged with (A) 5×10^5^ (10–40x LD_50_, n = 5 per group) or (B) 5×10^6^ (100–400×LD_50,_ n = 10 per group) Sterne *B. anthracis* spores administered intranasally. Mouse survival was monitored for 10 days post-spore challenge.

To test the efficacy of the antibody response elicited by PA-Av-C3d_3_/IFA immunization, we compared the ability of the various immunogens to provide protection against intranasal Sterne strain *B. anthracis* spore challenge. Groups of mice were immunized i.p. with either Av in IFA (Av/IFA), PA-Av/IFA, PA-Av-Alum, or PA-Av-C3d_3_/IFA. 10-days post immunization, mice were given approximately 5×10^5^ anthrax spores administered intranasally (Figure 6A). Control mice (Av/IFA-immunized) survived for only 4-days following anthrax challenge. Forty percent of PA-Av/IFA immunized mice survived anthrax challenge, with most deaths occurring between day 4 and 5. PA-Av-Alum immunization provided slightly enhanced protection (60% survival) relative to the PA/IFA, with most deaths occurring at approximately 6 days after anthrax challenge. PA-Av-C3d_3_/IFA immunization was the most effective, showing 80% survival from *B. anthracis* spore challenge.

Finally, we asked whether the enhanced antibody response elicited by PA-Av-C3d_3_/IFA immunization (Figure 1 & 2) could protect mice from increased spore dose. Therefore, we challenged mice with a 10-fold increase (5×10^6^) in Sterne strain *B. anthracis* spores (Figure 6B). We observed that mice immunized with PA-Av-C3d_3_/IFA exhibited 90% survival, indicating that C3d is an effective adjuvant against high-dose spore challenge. PA-Av-Alum and PA-Av/IFA immunized mice showed only 40% and 30% survival, respectively. Taken together, these data suggest that incorporation of C3d confers increased vaccine efficacy relative to PA/IFA or PA/alum as measured by protection from lethal anthrax challenge.

## Discussion

To avoid a calamitous outcome from *B. anthracis* infection, the humoral immune system needs to respond rapidly and effectively to limit or prevent pathogenic effects of anthrax toxins. If administered prior to the symptomatic stage, antibiotics are effective in stemming disease. However, since these symptoms are nonspecific, effective prophylactic vaccines are necessary to prevent spore germination, block vegetative replication and/or neutralize exotoxin function. The currently licensed Biothrax™ vaccine consists of aluminum hydroxide-adsorbed culture supernatant material from toxigenic unencapsulated strains of *B. anthracis*. In response to the acknowledged threat of *B. anthracis* spore dissemination as a biowarfare agent, usage of Biothrax™ for active vaccination programs and for prospective stockpiling has accelerated. The resurgent need for an anthrax vaccine(s) has also refocused concerns regarding the specific efficacy and nonspecific reactogenicity associated with vaccination. In response to the stated need to develop a well characterized and efficacious vaccine that is also well tolerated [6], we generated and characterized a novel subunit vaccine that employs C3d as a natural adjuvant and PA as the target antigen for neutralizing antibodies. In comparison to PA-Av-Alum or PA-Av/IFA, injection of PA-Av-C3d_3_/IFA by various routes of administration induced a more rapid and augmented PA-specific antibody response. Production of effector antibodies of all IgG isotypes was sustained, and no evidence of pro-inflammatory cytokines was observed.

Second generation anthrax vaccines currently in clinical trials consist of PA adsorbed to aluminum hydroxide. Hence, the adjuvant is the same as that used in Biothrax™, but lot variability in PA content as well as residual toxicity are reduced. Protective antigen is an attractive neutralization target since binding, uptake, and cytosolic release of LeTx and EdTx are dependent upon PA. We found that coupling of C3d to PA and emulsion in IFA elicited a rapid PA-specific antibody response in adult and adolescent mice that was superior to the response elicited by equivalent amounts of PA adsorbed to alum or emulsified in IFA. All IgG isotypes were efficiently induced by PA-Av-C3d_3_/IFA, indicating that sufficient T cell help was recruited to promote class switch recombination and continued differentiation into plasma cell effectors. Correspondingly, C3d conjugation enhanced the generation of GC B cells and the coincident generation of Syn1^+^ plasma cells. Rechallenge with PA in saline induced similar titers of PA-specific antibody in mice receiving primary immunizations of PA-Av-C3d_3_/IFA or PA-Av-Alum. These results could indicate the equivalent generation and survival of PA-responsive B cells from the primary response. However, we find it more likely that the sustained levels of anti-PA titers at 6 weeks post-immunization with PA-Av-C3d_3_/IFA leads to rapid binding and clearance of PA by Fcγ receptor-bearing effector cells, resulting in reduced availability of antigen in PA-Av-C3d_3_/IFA–primed animals. Unlike PA-Av-Alum or PA-Av/IFA, PA-Av-C3d_3_/IFA immunization was also capable of inducing rapid and sustained IgG titers against domain 4 of PA. This is an important hallmark, as domains 2 and 4 cooperate to bind the anthrax receptor and thus are indispensable for anthrax toxicity [10]. These findings imply that PA-Av-C3d_3_/IFA should be effective against natural or engineered variants of *B. anthracis*.

Adjuvants are necessary to augment the immune response by promoting lymphocyte activation and preventing rapid clearance (“depot effect”) of the immunogen. Despite the fact that aluminum salts have been in use for decades and are the only approved vaccine adjuvants in the U.S., little is known about how they act. Although some proteins adsorb to aluminum hydroxide with high efficiency, several reports have shown rapid desorption *in vivo* [40]. Such findings argue against a strong depot effect and suggest that adjuvancy may be achieved by the release of concentrated antigen. Microbial pathogens activate the innate immune system by engaging Toll-Like Receptors (TLRs), resulting in cytokine release and the upregulation of costimulatory molecules on antigen-presenting cells. It has been observed that aluminum compounds can promote the maturation or activation of myeloid antigen-presenting cell subsets; however, since TLR signaling does not appear to be involved it is unclear how this occurs [40], [41], [42], [43]. By contrast TLR signaling is thought to be a major contributor to the immunostimulatory activity of complete Freund's adjuvant (CFA), consisting of paraffin oil and mycobacterial components. CFA efficiently promotes the maturation and activation of myeloid cells and thus promoting their ability to act as antigen presenting cells for T cells. By comparison, IFA also promotes the slow release of the immunogen, but nonspecific immunostimulatory activity is minimal or absent in most preparations. We chose IFA emulsion for the work presented here, since we sought to determine the antigen-specific adjuvant activity of C3d in the absence of other costimuli. Since emulsion of PA-Av in IFA generally did not elicit an antibody response that was significantly greater than that of PA-Av alone, it is evident that the adjuvancy effect of PA-Av-C3d_3_/IFA is mainly attributed to C3d rather than IFA. Nonetheless, parallel studies using PA-Av-C3d_3_ in saline would be necessary to formally address the role of IFA in potentiating the effect of C3d conjugation.

In contrast to aluminum hydroxide, the adjuvancy effect of C3d is better understood [35]. Soluble proteins generally do not activate the complement cascade, but need to be opsonized via antibody binding or in the context of local inflammation - as may occur with other adjuvants that activate innate immunity. C3d facilitates antigen retention by binding to CD21/35 on FDCs, which do not efficiently endocytose antigen and may protect bound antigen from proteolysis, thus creating a depot effect. In addition to focusing antigen to the B cell surface, C3d promotes B cell activation by co-aggregating the BCR and CD21, resulting in enhanced signaling via CD21-associated CD19. In addition, C3d fixation facilitates the uptake and presentation of antigen to recruit T cell help [27], [29]. It is known that CD19 and CD21 are crucial for GC formation and resultant memory B cell generation. Therefore, in addition to promoting the recruitment of PA-specific B cells into the primary response, C3d-conjugation promotes the generation of high affinity antibody-secreting cells and the maintenance of the memory B cell compartment.

Results of a phase I trial comparing Biothrax™ and rPA/aluminum hydroxide (rPA102) was recently reported [17]. Toxin neutralizing assays revealed similar efficacy with either vaccine and reduced reactogenicity with rPA102. However, PA-specific antibody titers were significantly reduced in subjects receiving rPA102. Extrapolating from these findings and our own studies, we conclude that PA-C3d conjugates or fusion proteins hold great promise as a next generation vaccine. Unlike Biothrax™ or rPA102, PA-Av-C3d_3_ appears to elicit rapid and sustained PA-specific antibody production when administered i.p., s.c. or i.m., suggesting that protection against anthrax can be achieved and maintained with a single vaccination. Altogether, our data suggests that PA-linked-C3d would be efficacious as both a preventative vaccine and for the rapid induction of neutralizing antibody upon acute anthrax exposure.
